# Some Untoward Consequences of Phimosis and of Circumcision

**Published:** 1910-12-31

**Authors:** Geo. H. Edington

**Affiliations:** Professor of Surgery, Anderson's College Medical School; Assistant Surgeon, Western Infirmary, Glasgow, &c., &c.


### HOSPITAL CLINICS.

### SOME UNTOWARD CONSEQUENCES OF PHIMOSIS AND OF CIRCUMCISION.

By GEO. H. EDINGTON, M.D., F.R.F.P.S.G., Professor of Surgery, Anderson's College Medical School; Assistant Surgeon, Western Infirmary, Glasgow, &c., &c.

In the practice of medicine and surgery one cannot but be struck with the way in which it becomes the fashion from time to time to attribute various morbid conditions or symptoms to disease of a particular organ. One of the most recent examples of this is the so-called "Appendix-dyspepsia," concerning which there has lately been so much discussion. Another example, by no means recent however, is the fact of phimosis having been looked upon as the cause of a variety of more or less remote ailments, such as spastic palsies, simulated hip-joint disease, muscular inco-ordination, convulsions.* The performance of circumcision was not, however, universally followed by relief from the symptoms supposed to depend on the abnormal tightness of the prepuce, and opinion as to the etiological relationship of phimosis to the ailments in question underwent a change, or at least a modification. Surgeons continued to perform the operation; but they were more chary of giving a glowing prognosis. In his recent work on the *Surgery of Children*, Kirmisson,† referring to Sayre's view that phimosis may cause reflex contractures and even paralysis, states that he has not seen any case which has convinced him of the truth of this statement. I may add that my experience is in agreement with that of the French surgeon.

No one can deny that there are some conditions which undoubtedly result from phimosis, and that in these cases circumcision will effect a cure on the principle "causa sublata, tollitur effectus." I do not intend to deal with all of these conditions. I wish merely to mention two cases in which phimosis caused obstruction to micturition, and in which surgical interference became a matter of utmost necessity. In recording these cases I would expressly state that such examples are by no means common.

### RETENTION OF URINE.

In March of this year (1910) I was asked by his medical attendant to see a gentleman aged eighty-four. The patient was the subject of congenital phimosis, and occasionally suffered from retention of urine during attacks of balanitis. On these occasions his doctor was in the habit of relieving him by the passage of a No. 4 rubber catheter. The present attack of retention commenced in the morning, and when the medical man saw him in the evening it was found that the catheter could not be introduced into the meatus.

There was no redundancy of the prepuce, but phimosis existed to an extreme degree, and the bladder was greatly distended. The preputial orifice was so tight that I could not get the point of a scissors-blade through it. After subcutaneous injection of novocaine and suprarenin, access to the glans was obtained by cutting down in the medial line of the dorsal surface of the prepuce. When the mucous layer had been penetrated some urine retained in the preputial sac escaped. The incision was then extended forwards and the orifice of the prepuce laid open. At first no appearance of a meatus could be seen, but very soon urine was ejected with considerable violence from a minute orifice, the stream continued till the patient had emptied his bladder, and his groans were exchanged for expressions of relief.

It was difficult to recognise the preputial orifice on account of its small size. Its edge was hard and gristly, but did not seem to be the seat of any acute inflammatory change. There was dense adhesion between the mucous layer of the prepuce generally and the glans, extending forwards on the dorsum and on the right to quite close to the meatus: the corona could be exposed only in the ventral portion of the left half of the glans, and the prepuce could not be stripped back. The mucous layer was stitched to the skin at the margins of the incision. Recovery was uneventful.

Phimosis may cause considerable difficulty in urination, and the difficulty may be greatly increased by the swelling accompanying balano-posthitis; but it seems to me that actual retention of urine occurring in the absence of marked inflammatory swelling of the parts is distinctly uncommon. At any rate, I had not before seen an instance of it. The condition of affairs is comparable to what one is familiar with in the case of urethral stricture.

### PERINEAL PERI-URETHRAL ABSCESS.

In March 1905 a lad aged twelve, the subject of very tight phimosis, was admitted to the Western Infirmary under the care of Sir Hector Cameron, to whom I am indebted for permission to record the case. There was a history of pyuria and elevated temperature of four days' duration. There were signs of extravasation in the perineum, extending forwards into the posterior part of the scrotum. Circumcision was performed, and the perineum was incised in the median line, giving vent to pus, but without opening the urethra. The meatus when exposed by circumcision was seen to be atresic. After his return to bed he micturated before he had recovered consciousness, and at the close of the act pus was observed to come away in the urine. He made a good recovery, and when I saw him about a year ago he had no trouble with his urinary organs.

These two cases are interesting as extreme results of obstruction. In the second the obstruction was not complete; but it had been sufficient to act on the wall of the urethra in a way similar to what

* White and Martin's *Genito-Urinary Surgery and Venereal Diseases*. Fifth edition. London. 1902. Pp. 8.

† *Handbook of the Surgery of Children*. Translated by J. Keogh Murphy. London. 1910. Pp. 141.

occurs in the case of tight stricture of the urethra. In both cases there was some atresia of the meatus; but in neither was this sufficient to produce complete obstruction, and in the first it was quite evident that the retention was due entirely to the condition of the preputial orifice.

### Circumcision.

The frequency with which circumcision is performed on the children of non-Jewish parents has almost ceased to be a matter for comment. Infants are being constantly brought to the out-patient department of the Royal Hospital for Sick Children in Glasgow for circumcision, although there may be no phimosis, and the extent to which the custom has spread may be appreciated from the remark made by a young mother when I told her that her child did not require the operation: "I thought," said she, "all boys were circumcised." It is well, in view of this frequency, to bear in mind a post-operative condition which I do not think has received sufficient attention. I refer to acquired stenosis of the urethral meatus.

### Post-operative Meatal Stenosis.

Such stenosis is the result of superficial ulceration of the lips of the meatus, the ulceration being caused by irritation of the exposed meatus by the child's clothing. The fact that it is hardly ever observed in the children of well-to-do parents shows that with care it may be prevented; but in the majority of hospital cases cleanliness is not an outstanding virtue, and it is in these cases usually that this troublesome result is found.

The usual sequence of events is that the patient is brought back to the hospital, in the course of two or three weeks after circumcision, with the story that he seems to have pain and difficulty in passing urine. Examination of the glans shows moist scabbing occluding the meatus, the lips of which are more or less ulcerated. Ultimately healing is followed by contraction of the meatus.

In circumcision cases treatment may be directed to prevent this ulceration. Such treatment consists in frequent bathing and in covering the part with vaseline or some unirritating ointment. If ulceration has occurred, the same treatment may be adopted, and when the ulcerative condition has been cured the meatus may be slit downwards into the frænum, and the cut edges of the urethral mucous membrane united by suture to the skin of the frænum. It may be objected by some that the condition is really a congenital stenosis which, as is well known, not infrequently accompanies phimosis; but if the precaution be taken of inspecting the meatus at the time when circumcision is done, it will be found that narrowing of a previously normal meatus may occur in the way I have mentioned.

### Constriction of Post-Coronal Sulcus by Hair.

I have quite recently observed a case in which, from want of attention, this unusual condition was present. I record it as a curiosity. Baby C., aged six months, was brought to the out-patient department of the Royal Hospital for Sick Children in November 1910 on account of great swelling of the glans of one day's duration. He had been circumcised at the age of three weeks, and no trouble had ensued till the present. The glans was enlarged to about the size seen in the adult. It was quite pale and showed no signs of venous congestion. A deep furrow was present behind the corona, and on retracting the skin of the penis there was observed in the furrow what looked like dark hairs wound round the organ. Examination under an anæsthetic confirmed this, and showed further that the hair, two or three ply, had actually produced a ring of ulceration, which on the ventral aspect had extended right through the frænum. The constricting hairs were divided with scissors and removed, and the mother was directed to bathe the parts frequently and to anoint with vaseline.

That this condition may arise independently of circumcision was demonstrated a week later, when a boy aged 7 was brought to the hospital on account of soreness of the penis of one week's duration. This boy was uncircumcised, but he had retracted the prepuce, which was shorter than usual, and in the exposed post-coronal sulcus was a constricting wisp of hair and wool. The constricting material had produced a ring of ulceration which extended round behind the corona and involved the frænum. The treatment adopted was the same as in the former case. In neither of the cases was there any suggestion of intentional ligation of the penis. The nature and bulk of the constricting material pointed rather to its having come either from the patient's clothing or from towels in common use. I mention these conditions of meatal stenosis and post-coronal constriction as examples of dangers to which the exposed glans is liable. Their prevention depends on proper care and cleanliness being seen to by the child's mother or nurse.

